# Vacancy-Induced Magnetism in Fluorographene: The Effect of Midgap State

**DOI:** 10.3390/molecules26216666

**Published:** 2021-11-03

**Authors:** Daozhi Li, Xiaoyang Ma, Hongwei Chu, Ying Li, Shengzhi Zhao, Dechun Li

**Affiliations:** 1 School of Information Science and Engineering, Shandong University, Qingdao 266237, China; 201912459@mail.sdu.edu.cn (D.L.); hongwei.chu@sdu.edu.cn (H.C.); Shengzhi_zhao@sdu.edu.cn (S.Z.); 2 Key Laboratory of Colloid and Interface Chemistry of Education Ministry, School of Chemistry and Chemical Engineering, Shandong University, Jinan 250100, China; yingli@sdu.edu.cn

**Keywords:** fluorographene, vacancy, magnetic moment, strain

## Abstract

Based on density functional theory, we have systematically investigated the geometric, magnetic, and electronic properties of fluorographene with three types of vacancy defects. With uneven sublattice, the partial defect structures are significantly spin-polarized and present midgap electronic states. The magnetic moment is mainly contributed by the adjacent C atoms of vacancy defects. Furthermore, the strain dependence of the bandgap is analyzed and shows a linear trend with applied strain. This defect-induced tunable narrow bandgap material has great potential in electronic devices and spintronics applications.

## 1. Introduction

Graphene has received widespread attention for its unique chemical or physical characteristics and exhibiting great potentials in optics, spintronics, and optoelectronics since its discovery [1,2,3,4,5,6,7]. However, the gapless band structure, which fails to switch current “on” and “off”, obstructs its applications in electronics such as field-effect transistors (FET). In order to overcome this obstruction, to date, diverse strategies have been proposed to open the zero bandgap and one of the effective schemes is chemical functionalization [8,9,10,11,12,13]. Functional graphene, including graphene oxide and halogenated graphene, has been proved to possess extraordinary properties as well as an expected tunable bandgap [14,15,16,17,18,19]. For example, by halogen (F, Cl, Br, I) doping, halogenated graphene is suggested to be capable to regulate the bandgap in a wide range and also could enhance the reaction kinetics of the Li–S cathode, leading to a high-performance lithium battery [20,21]. As the F atom possesses a higher electronegativity than other halogen atoms, fluorinated graphene (CF*_x_*), a typical type of halogenated graphene, has been verified to be more stable than other types, and its properties are strongly dependent on the degree of fluorination [22,23,24]. By modulating F/C ratios, graphene, a nonmagnetic semimetal, can be transformed into a nonmagnetic/magnetic semiconductor/insulator [25]. With an F atom attached to each C atom, fluorographene (fully fluorinated graphene) was reported to be a high-quality insulator (resistance >10 GΩ at room-temperature) with a wide optical bandgap (3.8 eV), large negative magnetic resistance (a factor of 40 in 9T field), and remarkable mechanical strength, showing great potential in the electronic applications [26,27].

During the past two decades, many efforts have been made to fabricate fluorographene [27,28,29,30]. In 2010, Cheng, et al. reported the synthesis of graphene fluoride by reacting graphite and fluorine gas. They demonstrated that the band structure and conductivity of CF*_x_* were reversible by fluorination or reduction reactions [27]. After that, in 2011, Jeon and his collaborators produced fluorographene with the treatment of graphene with xenon difluoride (XeF_2_) and pave the way to develop graphene-based semiconductors by the direct chemical fluorination method [28]. Recently, the successful thermal exfoliation of fluorinated graphene at room temperature indicated the feasibility of producing large-scale fluorographene [30]. However, considering the high temperature involved in the fluorination processes, structural defects, especially vacancy defects may appear and deteriorate the performance of structures such as magnetic momentum and conductivity, which has an important impact on the magnetic and electronic applications [27,31]. Many calculations also show that defects will have a great influence on electromagnetic properties of two-dimensional materials [32,33]. Therefore, it is of great meaning to explore the theoretical mechanism of vacancy defects and deeply understand the defects’ influence on structural performance. In this article, we studied fluorographene with three types of vacancy defects, including single F atom vacancy (V_F_-fluorographene), single C–F vacancy (V_sCF_-fluorographene), and double CF vacancy (V_dCF_-fluorographene) via first-principle theory. The ab initio molecular dynamics (MD) simulations were performed to estimate the thermodynamical stability. The spin-charge density was analyzed to figure out how magnetic moment induced by vacancy defects. Furthermore, band structures, as well as the density of states, are also investigated. Although fluorographene is an insulator with a large bandgap, it can be transformed into a semiconductor by introducing appropriate vacancy defects and the bandgap can be tuned by the external strains.

## 2. Results and Discussion

**Figure 1:**
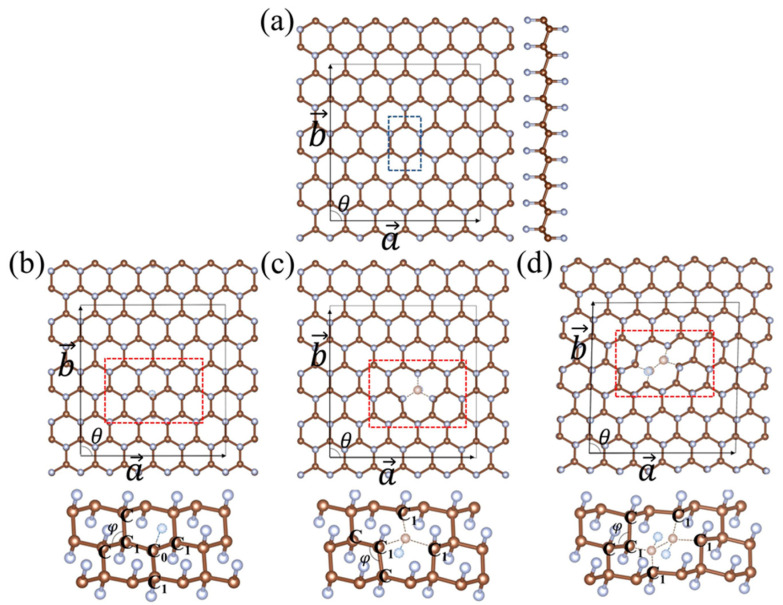
The 5 × 3 supercell of (**a**) fluorographene, and three types of vacancy defects: (**b**) fluorographene with F vacancy (V_F_-fluorographene), (**c**) fluorographene with single C–F vacancy (V_sCF_-fluorographene), and (**d**) fluorographene with double C–F vacancy (V_dCF_-fluorographene). The brown and light-grey spheres represent C and F atoms, respectively. The blue rectangular in (**a**) is the unit cell of fluorographene, while the red rectangular in (**b**–**d**) represent the areas greatly impacted by vacancy defects.

To avoid the interactions between defects, 5 × 3 supercell of fluorographene is established. We chose three typical vacancy structures (single F, single C–F, and double C–F vacancy) by referring to the vacancy defect structures in other functional graphene [14,15]. Figure 1 displays the structures of V_F_-, V_CF_- and V_dCF_-fluorographene, respectively.

The C–F bond length (1.384 Å) and angle between adjacent C–C bonds (110.8°) are in good agreement with the previous calculation (1.37 Å/111°) [34]. With an F absence, the C_0_ atom in V_F_-fluorographene connects to three nearest neighbor C_1_ atoms, and there is a slight distortion in the lattice, especially in the red rectangular where the lattice is greatly perturbed by the vacancy defect. The buckling height of the C_0_ atom decreases remarkably due to the enhancement of the C_0_–C_1_ bonding strength. Similarly, for the V_sCF_- and V_dCF_-fluorographene, the bond strength of C_1_–C enhanced since C_1_ atoms move close to their adjacent C atoms. The vacancy defects break the original symmetry of fluorographene. V_F_- and V_sCF_-fluorographene fluorographene shows mirror symmetry, while V_dCF_-fluorographene shows central symmetry.

The structural data of fluorographene and fluorographene with vacancy defects are summarized in Table 1. The decrease of the average bond length $d_{C_{1}-C}$ in all these structures confirms the enhancement of C_1_–C bonding strength. With the F defect, the average C_1_–F bond length $d_{C_{1}-F}$ in V_F_-fluorographene show an unexpected increase, which is quite different from V_sCF_- and V_dCF_-fluorographene. This shows that compared to fluorographene, the interaction between C_1_ and F atoms of V_F_-fluorographene is weaker, while that of V_sCF_- and V_dCF_-fluorographene are stronger. The deviation of lattice angle θ in V_dCF_-fluorographene demonstrates that the deformation caused by the double C–F vacancy is stronger than others

**Figure 2:**
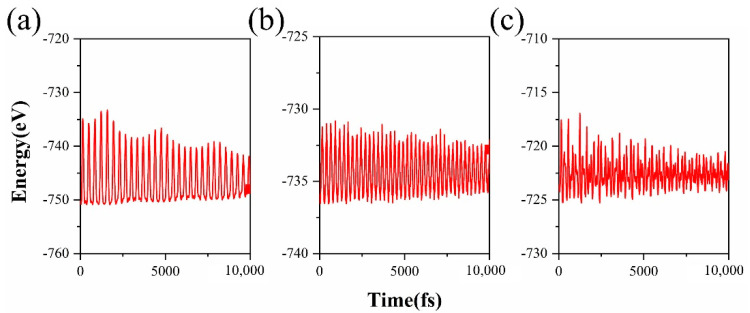
Variation of the total energy of (**a**) V_F_-fluorographene, (**b**) V_sCF_-fluorographene, and (**c**) V_dCF_-fluorographene during the AIMD simulation at room temperature. The inset pictures are the atomic configurations after 10 ps.

The formation energies of the fluorographene with vacancy defects are shown in Table 1. Even though the energies of vacancy configurations are slightly larger than that of fluorographene, the small deviations (less than 40 meV/atom) suggest that V_F_-, V_sCF_-, and V_dCF_-fluorographene could be stabilized at nonequilibrium conditions. We performed the ab initio molecular dynamics (AIMD) simulation to verify the thermodynamic stability of fluorographene with vacancy defects at room temperature (300 K). The results are shown in Figure 2. As the variations in the total energies are within 0.15 eV/atom and the atomic structures maintain well during AIMD simulation for 10 ps, V_F_-, V_sCF_-, and V_dCF_-fluorographene are predicted to be thermodynamically stable at room temperature.

By absorbing the F atom, the depletion of the local π bond causes charge transfer in fluorographene. Since F atom possesses a higher electronegativity than C atom, electrons transfer from the C atom to its connected F atom, indicating that C–F is a polar covalent bond. The calculated charge of the C atom and F atom, obtained by the Hirshfeld-based method [9], are +0.48, −0.48, respectively. Sounding F atom vacancy, C_0_ atom remains ~4 by maintaining its unpair electron instead of reducing its electron passes to F atom, while the charge sharing of C_1_–F bonds adjacent to the vacancy has some little deviations. The charge sharing of the C–F bond is closely related to the third-order nonlinear optical response [35]. The Bader charge of nearest C–F bonds in V_sCF_- and V_dCF_-fluorographene also has been investigated and it is convinced that the charge transfer scheme will be affected by inducing vacancy. This result is in agreement with the change of bond strength shown in Table 1.

**Figure 3:**
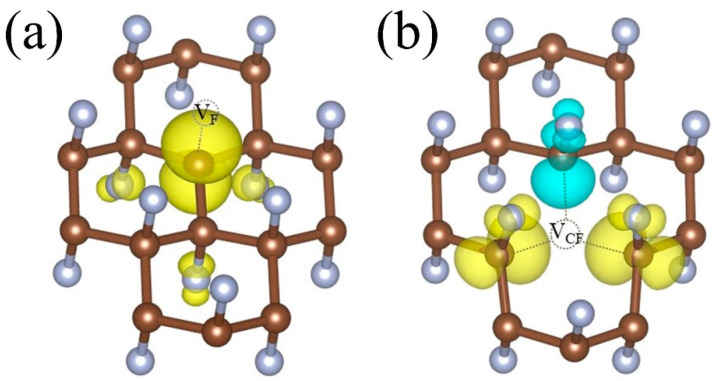
The spin-charge densities $n_{↑}\left(r\right)-n_{↓}\left(r\right)$ in (**a**) V_F_-fluorographene and (**b**) V_sCF_-fluorographene. Yellow and blue colors represent spin-up and spin-down isosurface, respectively.

To figure out whether vacancy can induce magnetic moment in fluorographene, we analyzed the spin density of defects structures and found that both V_F_- and V_sCF_-fluorographene are magnetic and hold 1μ_B_ magnetic moment, whereas V_dCF_-fluorographene is nonmagnetic. This is consistent with the reports that only fluorinated graphene with uneven F atoms in the double sides is magnetic and can be explained by Lieb’s theorem [36]. Figure 3 demonstrates the spin densities of V_F_- and V_sCF_-fluorographene. It is obvious that the magnetic moment of V_F_-fluorographene mostly comes from the C_0_ atom (0.72 μ_B_) nearest to F vacancy, while that of V_sCF_-fluorographene is mainly provided by C_1_ atoms (0.48 μ_B_, 0.49 μ_B_, and −0.29 μ_B_) next to the C–F vacancy. The discrepancy can be explained by the asymmetry structure as a consequence of vacancy defects. Furthermore, the three F atoms adjacent to C_1_ atoms have nonnegligible contributions: 0.06 μ_B_ each F aligned ferromagnetically for V_F_-fluorographene, (0.09 μ_B_, 0.09 μ_B_, −0.06 μ_B_) for V_sCF_-fluorographene.

**Figure 4:**
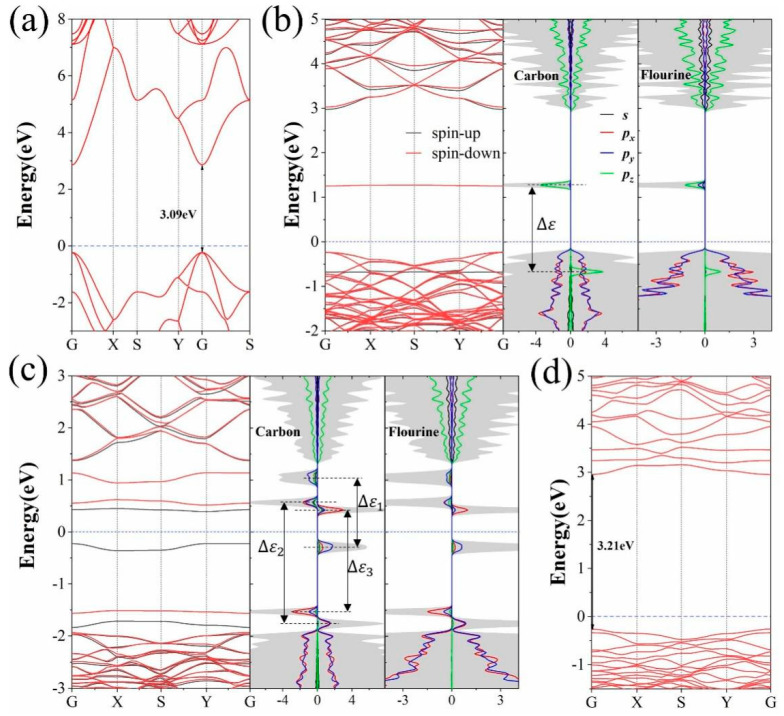
The band structure of (**a**) fluorographene (**b**) V_F_-fluorographene (**c**) V_sCF_-fluorographene (**d**) V_dCF_-fluorographene. For the magnetic structures, the PDOS is shown; the black line represents the spin-up and the red line represents the spin-down; in PDOS, the black, red, blue, and green represent the *s*, *p_x_*, *p_y_*, *p_z_* orbital of C and F, respectively. The shadow represents the total density of states and the light blue dot represents the fermi-level. $∆\varepsilon =\varepsilon_{↑}-\varepsilon_{↓}$, defined as the difference between spin-up and spin-down, is the exchange splitting energy.

The electronic properties of fluorographene and fluorographene with vacancy defects are investigated to further understand the origin of magnetism. The results are shown in Figure 4. It is revealed that fluorographene is an insulator with a bandgap of 3.09 eV, which is in good agreement with early reports (3.10 eV) [23], and V_dCF_-fluorographene has a 3.21 eV bandgap. Both fluorographene and V_dCF_-fluorographene have no spin splitting (see Figure S1 in Supplementary Materials). With uneven F atoms in the double sides induced by a single F vacancy defect, midgap states appear and V_F_-fluorographene is a semiconductor with a direct bandgap of 1.48 eV. The bandgap is tuned by the arise of the spin splitting. The flatness of the midgap band means that electrons are strongly localized and the localizations mainly come from *p_z_* orbital of both C and F atoms, conforming to the characteristics of defects states. This defect level is caused by spin-down states only. Different from V_F_-fluorographene, in V_sCF_-fluorographene, the valence band maximum (VBM) and conduction band minimum (CBM) are mainly contributed by *p_x_* and *p_y_* orbitals of spin-up states. The bandgap decrease to 0.61 eV and V_sCF_-fluorographene is a semiconductor with a direct bandgap. For both V_F_ and V_sCF_-fluorographene, because of the exchange splitting of the defect states, the *p* orbital of C and F atoms is hybridized and produces exchange split bonding and antibonding states which are the origin of the induced magnetic moment near vacancy.

**Figure 5:**
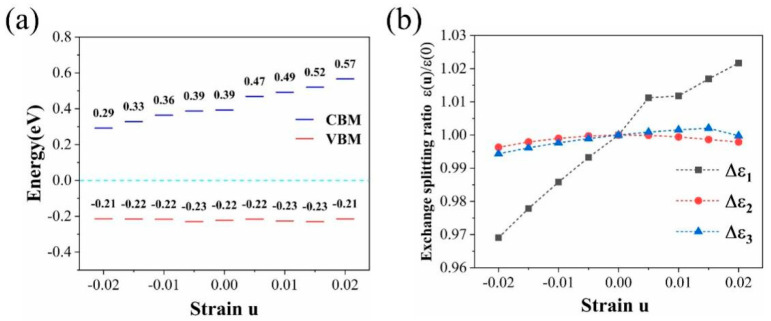
(**a**) Bandgap and (**b**) the exchange-splitting for V_sCF_-fluorographene with the uniaxial strain applied in zigzag direction.

Considering strain is an inevitable factor during fabrication, we further examined the strain dependence of bandgap and exchange-splitting of V_sCF_-fluorographene. The results are shown in Figure 5. By applying strain from −0.02 to 0.02 in zigzag direction, the bandgap of V_sCF_-fluorographene shows a linear increase from 0.51 eV to 0.78 eV. It should be noted that the position of the VBM changes infinitesimally, while the CBM changes greatly. The strain dependences of exchange-splitting are shown in Figure 5b. It is worth mentioning that the defect states related to $∆\varepsilon_{1}$, which is mostly contributed from *p_y_* orbitals, has a significant increase with applied strain, while that of $∆\varepsilon_{2}$ and $∆\varepsilon_{3}$ change slightly. The changes of exchange-splitting eventually tune the bandgap of V_sCF_-fluorographene. In contrast to the bandgap, the magnetic moment of V_sCF_-fluorographene remain 1μ_B_ magnetic moment and show no obvious change with the uniaxial strain applied in the zigzag direction (see Table S1).

## 3. Method

Density functional theory (DFT) calculations are completed using the Vienna ab initio simulation package (VASP). The projector augmented wave (PAW) method and generalized gradient approximation (GGA) are performed to describe the core valence interaction and exchange-correlation [37,38,39]. A grid of 5 × 5 × 1 kpoints generated by Monkhorst–Packscheme method [40] is used for the defect fluorographene and the cutoff energy is set as 500 eV to verify the accuracy of energy convergence. All structures are relaxed to maximum atomic forces allowance of 1 × 10^−2^ eV/Å and total threshold energy of 1 × 10^−7^ eV. Spin polarization is considered in all of the calculations by setting ISPIN = 2, and the non-collinear version of VASP is used to complete the magnetic calculation. The thickness of the vacuum layer between the monolayers is set as 15 Å to avoid the spurious interlayer interaction in the out-of-plane direction.

## 4. Conclusions

In summary, we studied the magnetic and electronic properties of fluorographene with three types of vacancy defects by using first-principle calculations. Our results indicate that all the three structures: V_F_-, V_sCF_-, V_dCF_-fluorographene are stable at room temperature. Due to the uneven F atoms in the double sides caused by defects, V_F_-, V_sCF_-fluorographene has been proved to be magnetic and possesses 1μ_B_ magnetic moments. The magnetic moment is mainly contributed by the adjacent C atoms of vacancy defects. We also investigated the strain dependence of V_dCF_-fluorographene, and it is found that the bandgap, as well as exchange-splitting energy, can be tuned by applied strain, especially the position of the valence band. The study of fluorographene paves the way for fabricating and analyzing fluorographene-based devices.

## Figures and Tables

**Table 1:** The structural data including lattice information, average bond length ($d_{C_{1}-C}$ and $d_{C_{1}-F}$), average angle between adjacent C_1_–C ($\varphi_{C-C_{1}-C}$) and formation energy of fluorographene, V_F_-, V_sCF_-, and V_dCF_-fluorographene. C_1_ is the nearest neighbor C atoms of vacancy defects.$\;E_{f}=(E_{total}-mE_{C}-nE_{F})/n_{tot}$, where $E_{C}$ and $E_{F}$ are the energy of C and F atom obtained from diamond and $F_{2}$, respectively. The m and n represent the numbers of the C and F atoms.

	$d_{C_{1}-C}\left(Å\right)$	$d_{C_{1}-F}\left(Å\right)$	$\varphi_{C-C_{1}-C}\left({}^{\circ}\right)$	a(Å)	b(Å)	θ(°)	$E_{f}(\mathrm{eV})$
fluorographene	1.575	1.384	110.807	13.015	13.525	90.00	−0.862
V_F_-fluorographene	1.518	1.412	113.828	12.933	13.442	90.00	−0.840
V_sCF_-fluorographene	1.52	1.342	109.363	13.035	13.497	90.01	−0.828
V_dCF_-fluorographene	1.523	1.361	107.185	12.931	13.254	88.47	−0.823

## Data Availability

The data presented in this study are available on request from the corresponding author. The data are not publicly available due to ensure the security of data.

## Supplementary Materials

The following are available online, Figure S1: The band structures and DOS of fluorographene and VdCF-fluorographene, Table S1: Magnetic moment of VsCF-fluorographene with the uniaxial strain [23].

## Sample Availability

Samples of the compounds fluorographene are available from the authors.
