# Supplementary material for: Vacancy-Induced Magnetism in Fluorographene: The Effect of Midgap State
Source: Molecules. 2021 Nov 3;26(21):6666. doi: 10.3390/molecules26216666 (PMC8587424; doi:10.3390/molecules26216666)
Supplement: Supplementary file 1 [file molecules-26-06666-s001.zip › molecules-1403938-supplementary.pdf]

SUPPLEMENT INFORMATION

For

Vacancy-induced Magnetism in Fluorographene: The effect of midgap state

Daozhi Li [1], Xiaoyang Ma [1,*], Hongwei Chu [1], Ying Li [2], Shengzhi Zhao [1] and Dechun Li [1,*]

1 School of Information Science and Engineering, Shandong University, Qingdao 266237, China
2 Key Laboratory of Colloid and Interface Chemistry of Education Ministry, and School of Chemistry and Chemical Engineering, Shandong University, Jinan 250100, China
* Correspondence: mxyacademic@sdu.edu.cn and dechun@sdu.edu.cn

1. The band structures and DOS of fluorographene and V_{dCF}-fluorographene

The DOS and PDOS for fluorographene and V_{dCF}-fluorographene are shown in Fig S1, in fluorographene, the valence band maximum (VBM) is mainly contributed by p_x by p_y, and conduction band minimum (CBM) is mainly contributed by p_z orbitals. It is found that fluorographene has no spin splitting and show nonmagnetic properties, which is consistent with the previous report[1], and so is V_{dCF}-fluorographene.

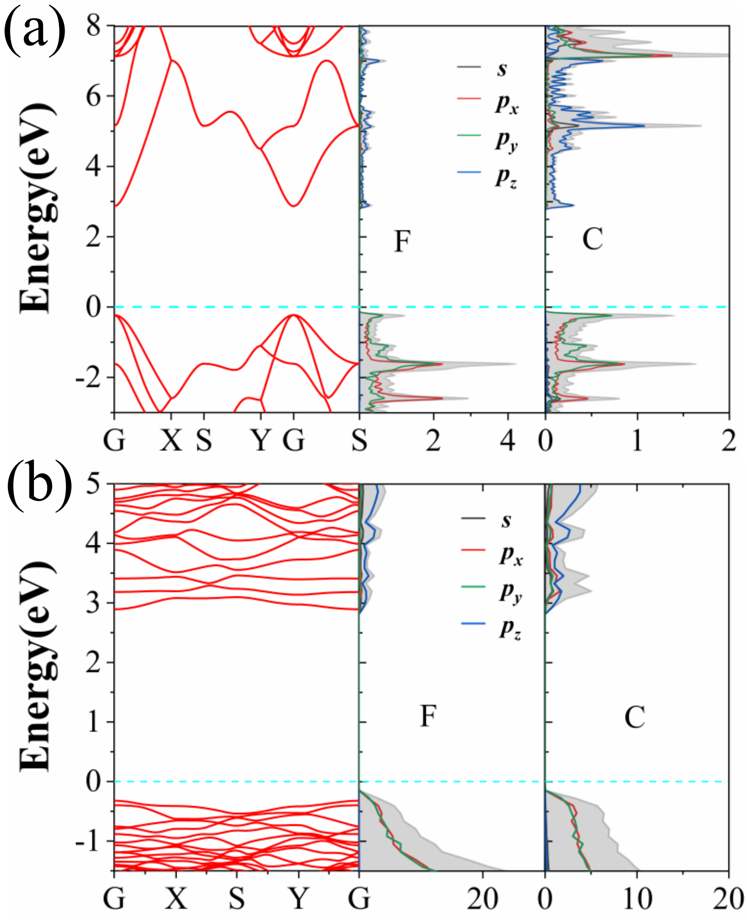

Figure S1. The band structure of (a) fluorographene (b) V_{dCF}-fluorographene. The black line represents the spin-up and the red line represents the spin-down; in PDOS, the black, red, blue, and green represent the *s*, p_x, p_y, p_z orbital of C and F, respectively. The shadow represents the total density of states and the light blue dot represents the fermi-level.

2. Magnetic moment of V_{sCF}-fluorographene with the uniaxial strain

With the uniaxial strain applied in the zigzag direction, the magnetic moment of V_{sCF}-fluorographene remain 1μ_B magnetic moment, thus, we can say that the strain has no obvious effect on the magnetic moment of the system. The details are listed in Table S1.

Table S1. The magnetic moment for V_{sCF}-fluorographene with the uniaxial tensile strain **u** applied in zigzag direction.

Strain **u**	-0.02	-0.015	-0.01	-0.005	0	0.005	0.01	0.015	0.02
Mag(μ_B)	1	1	1	1	1	1	1	1	1
